# Lucky Luke in the Cath lab? Use of a snare catheter to optimize positioning in transcatheter tricuspid valve replacement: a case report

**DOI:** 10.1093/ehjcr/ytag365

**Published:** 2026-05-14

**Authors:** Carolina Göttsche Esperança Clara, Muhammed Gerçek, Felix Rudolph, Mohammad Kassar, Kai P Friedrichs

**Affiliations:** Department of General and Interventional Cardiology/Angiology, Herz- und Diabeteszentrum NRW, Ruhr-Universität Bochum, Georgstraße 11, 32545 Bad Oeynhausen, Germany; Department of General and Interventional Cardiology/Angiology, Herz- und Diabeteszentrum NRW, Med. Fakultät OWL (Universität Bielefeld), Georgstraße 11, 32545 Bad Oeynhausen, Germany; Department of General and Interventional Cardiology/Angiology, Herz- und Diabeteszentrum NRW, Ruhr-Universität Bochum, Georgstraße 11, 32545 Bad Oeynhausen, Germany; Department of General and Interventional Cardiology/Angiology, Herz- und Diabeteszentrum NRW, Med. Fakultät OWL (Universität Bielefeld), Georgstraße 11, 32545 Bad Oeynhausen, Germany; Northwestern University Feinberg School of Medicine, 420 E Superior St, Chicago, IL 60611, USA; Department of General and Interventional Cardiology/Angiology, Herz- und Diabeteszentrum NRW, Ruhr-Universität Bochum, Georgstraße 11, 32545 Bad Oeynhausen, Germany; Department of General and Interventional Cardiology/Angiology, Herz- und Diabeteszentrum NRW, Med. Fakultät OWL (Universität Bielefeld), Georgstraße 11, 32545 Bad Oeynhausen, Germany; Northwestern University Feinberg School of Medicine, 420 E Superior St, Chicago, IL 60611, USA; Department of General and Interventional Cardiology/Angiology, Herz- und Diabeteszentrum NRW, Ruhr-Universität Bochum, Georgstraße 11, 32545 Bad Oeynhausen, Germany; Department of General and Interventional Cardiology/Angiology, Herz- und Diabeteszentrum NRW, Med. Fakultät OWL (Universität Bielefeld), Georgstraße 11, 32545 Bad Oeynhausen, Germany; Universitätsklinik für Kardiologie, Inselspital, Freiburgstraße 18, 3010 Bern, Switzerland; Department of General and Interventional Cardiology/Angiology, Herz- und Diabeteszentrum NRW, Ruhr-Universität Bochum, Georgstraße 11, 32545 Bad Oeynhausen, Germany; Department of General and Interventional Cardiology/Angiology, Herz- und Diabeteszentrum NRW, Med. Fakultät OWL (Universität Bielefeld), Georgstraße 11, 32545 Bad Oeynhausen, Germany

**Keywords:** Tricuspid valve regurgitation, Tricuspid valve replacement, Snare catheter, Case report

## Abstract

**Background:**

Transcatheter tricuspid valve replacement (TTVR) has emerged as a therapeutic option for patients with severe tricuspid regurgitation (TR) who are unsuitable for transcatheter edge-to-edge repair (T-TEER). While procedural outcomes continue to improve, device delivery remains a critical step and may be particularly challenging in complex anatomies.

**Case summary:**

We report on a 75-year-old patient with heart failure and severe TR of mixed aetiology with a history of cardiac resynchronization therapy (CRT). Surgical repair was deemed prohibitive due to high surgical risk, and transcatheter edge-to-edge repair was unfavorable, given the large coaptation gap. According to the Heart Team decision, TTVR using the Evoque system was planned. Intraprocedurally, conventional manoeuvres failed to achieve appropriate device positioning and delivery into the right ventricle. By employing a snare catheter and a steerable sheath, the delivery could be stabilized and guided into the correct position. This strategy ensured successful valve deployment at optimal depth, resulting in excellent valve function with only minimal paravalvular leakage.

**Discussion:**

Even with careful preprocedural planning with computed tomography, intraprocedural device delivery during TTVR can be challenging and result in procedural failure. The present case highlights the feasibility of combining a snare catheter with a steerable sheath to optimize device depth positioning and delivery, thereby ensuring procedural success in patients with challenging anatomies. Broader awareness and dissemination of such adjunctive techniques may expand the applicability of TTVR in anatomically challenging scenarios.

Learning points

- Complex anatomy due to pacemaker leads and large ventricles may make TTVR prosthesis deployment difficult.
- The use of a snare catheter for anterograde valve repositioning is a feasible technique for achieving optimal valve implantation depth.

## Introduction

Tricuspid regurgitation (TR) is an increasingly recognized valvular disease associated with heart failure, dyspnoea, and peripheral oedema, leading to impaired quality of life and poor prognosis.^1^ Affected patients are typically elderly and at high surgical risk, underscoring the need for interventional treatment options. Transcatheter tricuspid edge-to-edge repair (T-TEER) can address many patients; however, a considerable proportion remain untreated due to anatomical ineligibility.^2,3^ Transcatheter tricuspid valve replacement (TTVR), particularly with the Evoque system (Edwards Lifesciences, Irvine, CA), has emerged as a promising alternative and received CE mark as well as FDA approval. While implantation is carefully planned, procedural success may still be hampered by anatomical challenges not always predictable on computed tomography (CT). In contrast to T-TEER, which is often limited by leaflet morphology and coaptation geometry, TTVR is more determined by dimensions of the right atrium (RA), right ventricle (RV), tricuspid valve, and venous access.^4^ In particular, venous anatomy and the inferior vena cava (IVC)-RA angle can significantly complicate the procedure, even when device sizing appears adequate on CT. Device delivery remains the ‘elephant in the room’ for TTVR, emphasizing the need for novel adjunctive techniques to ensure optimal prosthesis positioning and expand the applicability of this therapy.

## Summary figure

**Figure:**
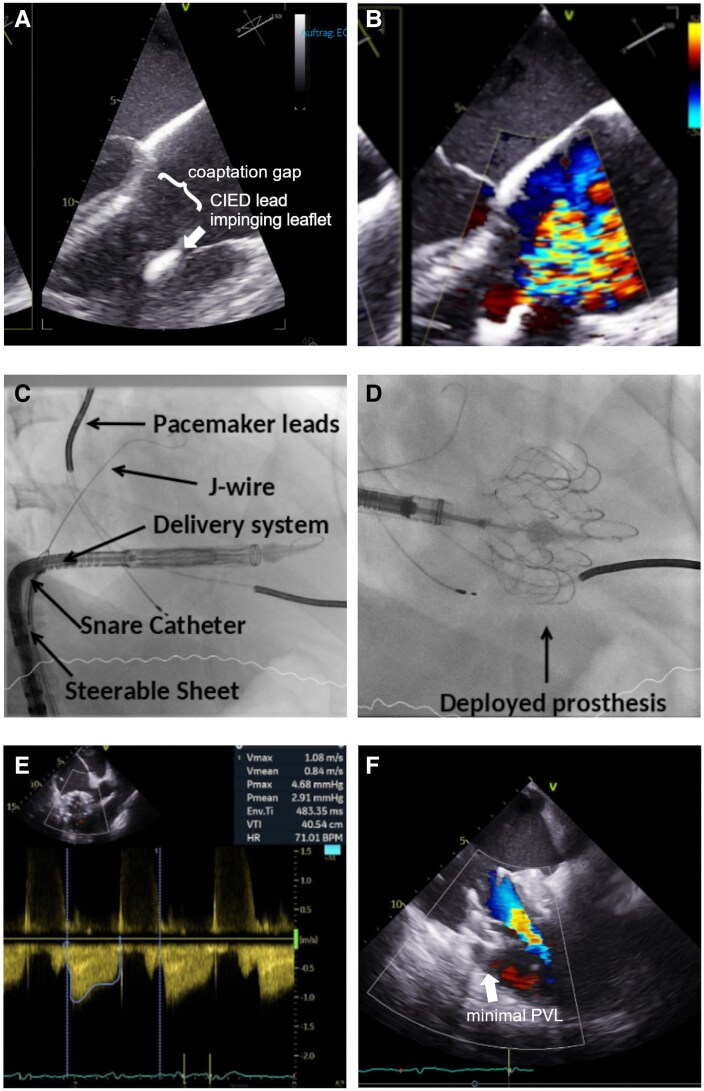


## Case presentation

**Figure 1:**
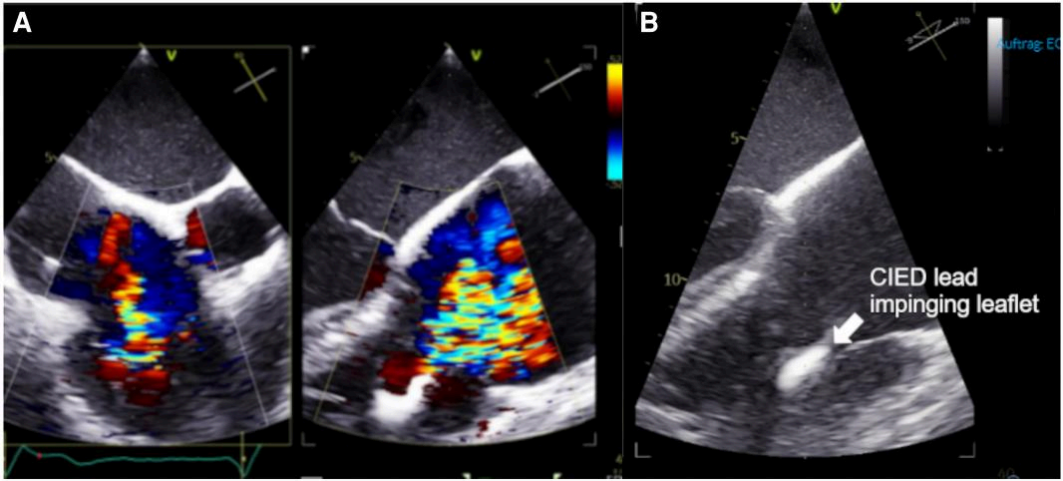
Image A shows severe tricuspid valve regurgitation of mixed etiology, partially due to leafleft impingement due to a cardiac implantable electric device (CIED, seen in Image B), in this case, an implantable cardioverter device.

We present a 75-year-old male patient with severe TR, a history of dilated cardiomyopathy, atrial fibrillation, and cardiac resynchronization therapy with defibrillator (CRT-D). At first, the patient was presented for implantable cardiac defibrillator (ICD) implantation due to reduced left ventricular ejection fraction (LVEF). Despite optimal medical therapy (OMT), LVEF did not improve significantly; therefore, in 2020, an upgrade to a CRT-D Device was pursued. Intraoperatively, no appropriate vessel was found to place the left ventricular lead. Hence, additional surgery to achieve left bundle branch area pacing was performed; however, postoperative imaging revealed LV lead dislocation. Respecting the patient’s wishes, no further intervention for LV lead retrieval and left bundle branch area pacing was attempted. In 2020, a mild TR was described. Follow-up echocardiography was performed yearly, with a progression to severe TR first discovered in 2024 (*Figure 1*). Despite the maximum tolerated dose of diuretics and OMT, the patient presented with dyspnoea equivalent to the New York Heart Association class III. Upon physical examination, severe peripheral oedema of the lower extremities, as well as ascites, were observed.

**Figure 2:**
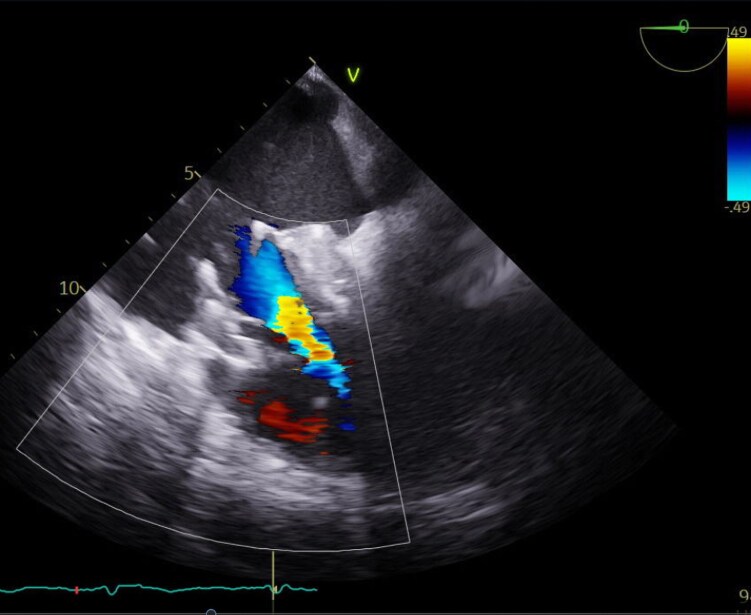
Computed tomography was performed to evaluate sizing parameters (A), as well as to obtain the projected working plane (B). Annular sizing was compatible with a 56 mm Evoque prosthesis. No interaction with the device lead was expected.

Transoesophageal echocardiography (TEE) revealed severe TR of mixed oetiology with lead impingement of the posterior leaflet with a large central coaptation deficit with a TR effective regurgitation orifice area (EROA) of 1.0 cm2. Moreover, right atrial and right ventricular dilation with a normal right ventricular ejection fraction of 59% and mildly reduced LVEF of 43% were described. Lead extraction was not considered a feasible option due to procedural risks and low expected impact on TR severity. Since the patient was deemed unfavourable for T-TEER because of the large coaptation deficit, a cardiac CT scan was performed to evaluate TTVR eligibility. A large coaptation deficit of 9 × 13 mm was confirmed, and the annulus size was measured. The TV annulus measured 53.9 mm in diastole and 51.5 mm in systole, as is shown in *Figure 2A*, rendering a 56 mm Evoque prosthesis suitable. The IVC-RA angle was 64°, RA height was 81.8 mm, and RV depth was 103.2 mm. Significant tethering was described. Regarding the device, there was no interaction to be expected, as the lead was near the posterior septal commissure with partial impingement of the posterior leaflet and minimal slack crossing the tricuspid valve annular (TVA) plane. The ICD coil was positioned <15 mm from TVA plane (*Figure 2B*). Considering the high symptomatic burden and relevant comorbidities (chronic kidney disease, type two diabetes, peripheral artery disease, and prostate cancer), the Heart Team decided on TTVR with an Evoque prosthesis, since the patient was opposed to surgical intervention.

The procedure was performed under general anaesthesia with angiographic and TEE guidance. The right femoral vein was accessed using a modified Seldinger technique, and a Safari-guide was advanced into the RV through an Agilis catheter. Afterward, the delivery system was advanced. Despite multiple manoeuvres, central orientation of the delivery system was not achieved. Hence, puncture of the left femoral vein was performed, and the right puncture site was cannulated with an 18F Cook sheath secured with a pre-placed Z-suture. Central orientation was attained; however, we were not able to generate sufficient implantation depth. A 20 mm balloon was positioned at the level of the upper IVC in an attempt to deflect the delivery sheath toward the RV and facilitate greater depth; however, this manoeuvre was unsuccessful. Next, an 18F Cook Sheath was used to access the right femoral vein, then a snare catheter (6F, 20 mm Amplatz GooseNeck; Medtronic), as well as an Agilis Sheath (medium curl 8.5F, Abbott) were advanced and positioned behind the Evoque delivery sheath, which was advanced through the left femoral site. Then the Evoque device was encircled, and the proximal shaft above the valve was snared. Fluoroscopic and echocardiographic imaging was used to ensure that there was no interaction with the CRT-D lead. The steerable Agilis Sheath was then employed to stabilize the delivery system and guide it deeper into the RV, thereby achieving sufficient depth for prosthesis implantation. A stepwise deployment of the prosthesis followed. Immediate postprocedural TEE revealed an excellent TTVR function with minimal central TR and trivial paravalvular leakage in the region of the device lead. A mean pressure gradient (MPG) of 2.8 mmHg was described under sedation.

**Figure 3:**
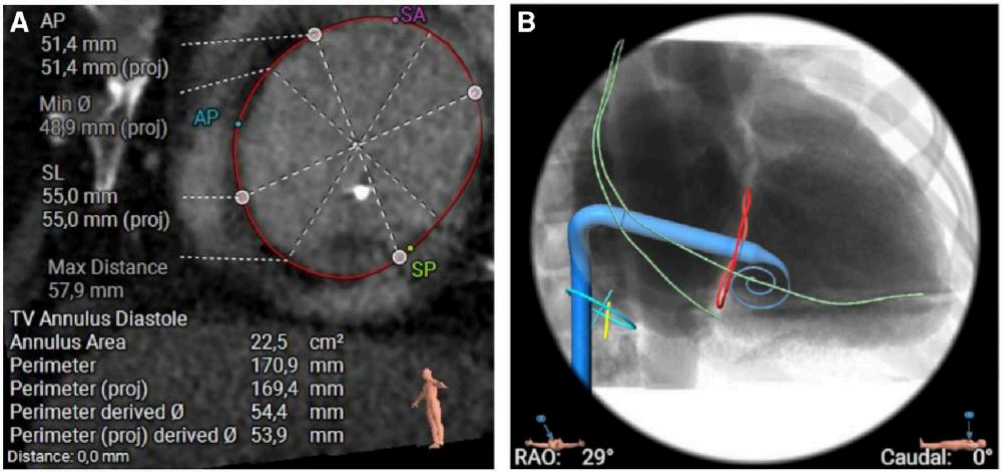
Transthoracic view of the triscupid valve after Evoque implantation showing the prosthesis in loco typico with minimal paravalvular leakage and a slightly increased mean pressure gradient (4.68 mmHg).

The patient had no vascular complications and was restarted on oral anticoagulation the day after TTVR. Post-operative transthoracic echocardiography revealed sufficient prosthesis function and an MPG of 4.68 mmHg (*Figure 3*). Due to the relatively high MPG, oral anticoagulation was switched from Apixaban to Phenprocoumon. At the 1-month follow-up, the patient reported reduced dyspnoea and reduced peripheral oedema under a moderate dose of diuretics. Echocardiography at the follow-up revealed trivial residual TR, minimal paravalvular leakage, and an MPG of 4 mmHg.

## Discussion

This case highlights a key consideration for the further development of TTVR strategies. TTVR offers an innovative treatment option for patients with TR in whom neither surgery nor T-TEER is deemed suitable. Accordingly, these patients often present with challenging anatomies, such as large coaptation gaps, severe leaflet tethering, or the presence of cardiac implantable electronic devices with impinged leaflets. Even with meticulous planning, intraprocedural device delivery may prove unexpectedly difficult.

In the present case, conventional manoeuvres failed to achieve correct valve positioning depth. An adjunctive off-label technique using a snare catheter in combination with a steerable sheath was therefore employed, allowing accurate positioning of the prosthesis and resulting in excellent valve function with only minimal paravalvular leakage.

Snare catheters are traditionally used to retrieve dislodged devices such as coronary stents, guidewires, or leadless pacemakers.^5^ More recently, they have also been applied to manage complications such as valve migration, situations that would otherwise require procedure abortion or surgical retrieval.^6^ In contrast, in our case, the snare catheter was proactively used to facilitate proper device positioning depth and deployment, thereby preventing the need for repositioning after valve release.

Beyond adjunctive catheter techniques, alternative access routes such as the transjugular approach have also been proposed to overcome anatomical limitations and facilitate more favourable device trajectories in selected patients.^7,8^ While still at an early stage of adoption, these strategies emphasize that device delivery remains the critical step for successful TTVR, warranting both technical innovation and interventionalist adaptability.

The successful outcome of our case underscores the importance of team experience and the ability to transfer skill sets from other interventional fields to challenging TTVR scenarios. Dissemination of such unconventional but effective techniques may contribute to expanding the safety and applicability of TTVR in anatomically complex patients.

## Conclusion

TTVR represents a promising option for patients with severe TR who are unsuitable for surgery or T-TEER. However, procedural success critically depends on safe and predictable device delivery. This case demonstrates that adjunctive manoeuvres, such as the off-label use of a snare catheter and steerable sheath, can facilitate accurate device positioning and prevent procedural failure after conventional positioning approaches have failed. Together with evolving alternative access strategies like the transjugular route, such techniques highlight the importance of continuous innovation and skill transfer to expand the applicability of TTVR in challenging patient populations.

## Data Availability

The data are available on request.

## Lead author biography

Dr. Carolina Göttsche Esperança Clara is a resident physician at the department of general and interventional cardiology/angiology at the Heart and Diabetes Center North Rhine-Westphalia, Germany.

## Notes

**Consent:** The authors confirm that written consent for submission and publication of this case report including images and associated text has been obtained from the patient in line with COPE guidance.
